# The indigenous vascular flora of the forest domain of Anela (Sardinia, Italy)

**DOI:** 10.3897/phytokeys.113.28681

**Published:** 2018-12-11

**Authors:** Emmanuele Farris, Michele Carta, Salvatore Circosta, Salvatore Falchi, Guillaume Papuga, Peter de Lange

**Affiliations:** 1 Dipartimento di Chimica e Farmacia – University of Sassari, Italy; 2 Agenzia forestale regionale per lo sviluppo del territorio e dell’ambiente della Sardegna, Forestas, Italy; 3 Provincia di Sassari, Settore Ambiente Agricoltura Nord Ovest, Italy; 4 Environmental and Animal Sciences, Unitec Institute of Technology, Private Bag 92025, Victoria Street West, Auckland 1142, New Zealand

**Keywords:** bioclimate, biodiversity, Mediterranean mountains, submediterranean, temperate

## Abstract

The importance of mountains for plant diversity and richness is underestimated, particularly when transition zones between different bioclimates are present along altitudinal gradients. Here we present the first floristic data for a mountain area in the island of Sardinia (Italy), which exhibits Mediterranean bioclimates at the bottom and temperate bioclimate at the top. We discovered a very high floristic richness, despite the fact that the number of endemic taxa is not high and the number of exclusive taxa is very low. Many of the detected taxa are at their range periphery and/or ecological margin. We conclude that climate transition zones in Mediterranean mountains and especially on islands are key areas regarding plant biodiversity and should be better investigated and protected.

## Introduction

Mountains are a critical landscape and ecosystem; they not only provide water for the lowlands but are a source of well-being and inspiration for numerous people (Korner 2004). The green ‘coat’ of the world’s mountains is composed of specialised biota, all nested in a great variety of microhabitats. Mountains biota are determined by a series of climatically different life zones over short elevational distances (Rahbek 1995, Korner 2000, Hemp 2002, Korner and Paulsen 2004), which often result in areas of high biodiversity of high conservation interest (Korner 2004). However, those areas are also under high threat regarding climate change, as it is expected that they experience drastic changes (Inouye 2008).

Mountain biodiversity can be studied at a multitude of scales in space, time and function (Molau 2004). Even though species richness is usually the focal component in nature conservation, genetic diversity within species is equally important. The small-scale distribution of species in the tropical Andes, as exemplified by the plant genera *Calceolaria* (Calceolariaceae) and *Bartsia* (Orobanchaceae), contrasts against the situation in high-latitude mountains, e.g. the Scandes, where species have wide ranges and many are circumpolar (Molau 2004). Several studies on alpine plants, based on molecular data, show that the intraspecific genetic diversity tends to increase with latitude, a situation brought about by glaciation cycles permitting repeated contraction-expansion episodes of species’ distributions (Abbott et al. 2000, Abbott and Brochmann 2003, Gamache et al. 2003, Holderegger and Abbott 2003, Lian et al. 2003, Abbott and Comes 2004). In tropical mountains, species distributions are geographically much narrower, often as a result of relatively recent, local speciation (Deshpande et al. 2001, Friar et al. 2001, Tremetsberger et al. 2003a, 2003b, Zhou et al. 2003). Thus, the classical decrease of genetic diversity observed from the equator toward the pole can eventually be blurred for mountain species. Actually, repeated contraction-expansion of species ranges has led rear edge populations to maintain some genetic diversity, therefore counterbalancing the effect of peripheral isolation (Hampe and Petit 2005). Conjointly, the high genetic differentiation between populations underlines the conservation relevance of those populations.

Mediterranean mountains represent an interesting case, because they often have a relic temperate-like bioclimate at their top (with no or little summer drought) in a context characterised by severe water deficit for at least two consecutive months at lower altitudes. Mediterranean mountains can therefore be considered as climatic islands, where plant diversity patterns are influenced by different factors (or in different ways) with respect to temperate and boreal mountains (Winkler et al. 2016). Furthermore, climatic and land-use changes have different effects on Mediterranean vs Boreal-Temperate mountains of Europe, being detrimental for the floral richness of the first and increasing the species richness of the second (Pauli et al. 2012). Considering that expected climatic trend is an increasing of temperature and a decrease of precipitation (mainly during spring) in Mediterranean mountains, whereas non-Mediterranean European mountains will not experience a reduction of annual and spring precipitation (Bravo et al. 2008), the urgency rises to monitor those mountains at the transition between Temperate and Mediterranean bioclimates. Moreover, before the middle of the century, the expected climatic changes will provoke the disappearance or strong reduction of a suitable habitat in the summit area, where most of the endemic and/or rare species are located (Benito et al. 2011). The most endangered habitats and species are those linked to water availability like streams, wet meadows and temporary ponds (Ghosn et al. 2010, Pérez-Luque et al. 2015). On islands, threats to mountain floras are even more acute compared to mainlands, because narrower spatial scales of habitats and the usually lower mountain altitudes (Vogiatzakis et al. 2016), led some species to have a relic distribution (Petit et al. 2005, Mayol et al. 2015, Fazan et al. 2017). Historical climatic fluctuations and associated ecological constraints are the basis of the fragmented distribution of Boreal-Temperate species on Mediterranean mountains (Mayol et al. 2015, Iszkulo et al. 2016) and determine the presence of plant refugia, climatically stable areas that constitute key areas for the long-term persistence of species and genetic diversity, especially at present and future decades given the threat posed by the extensive environmental change processes operating in the Mediterranean region. These refugia, including large Mediterranean islands, represent ‘phylogeographical hotspots’; that is, significant reservoirs of unique genetic diversity favourable to the evolutionary processes of Mediterranean plants (Médail and Diadema 2009).

The island of Sardinia, the second largest in the whole Mediterranean basin, was already known to have a prevalent Mediterranean bioclimate, with a temperate bioclimate in the two main massifs of the island, the Gennargentu (centre-eastern Sardinian, maximum elevation 1834 m a.s.l.) and the Limbara (north-eastern Sardinia, maximum elevation 1359 m a.s.l.) (Arrigoni 1968). Recent detailed bioclimate analysis (Canu et al. 2015) also showed that the only mountain chain of the island named Marghine-Goceano (located between the Limbara and the Gennargentu massifs, maximum elevation at Mt. Rasu 1259 m a.s.l.) is characterised by a temperate bioclimate (in the sub-Mediterranean variant) along the ranges summit. Although the mountain floras of the Gennargentu and Limbara are well documented (Veri and Bruno 1974, Arrigoni and Camarda 2015), floristic information about the Marghine-Goceano range is lacking (Valsecchi and Corrias 1966).

This paper goes some way to fill this knowledge gap by reporting on the indigenous flora of a forest domain located in the middle of the Marghine-Goceano range. Our aim was to provide a checklist of the flora located in this area to enable future characterisation of the biotic environment of this mountain area of Sardinia. This data will also allow the identification of target species to monitor and understand climate changes in the particular context of Mediterranean islands.

## Methods

### Study area

**Figure 1.**
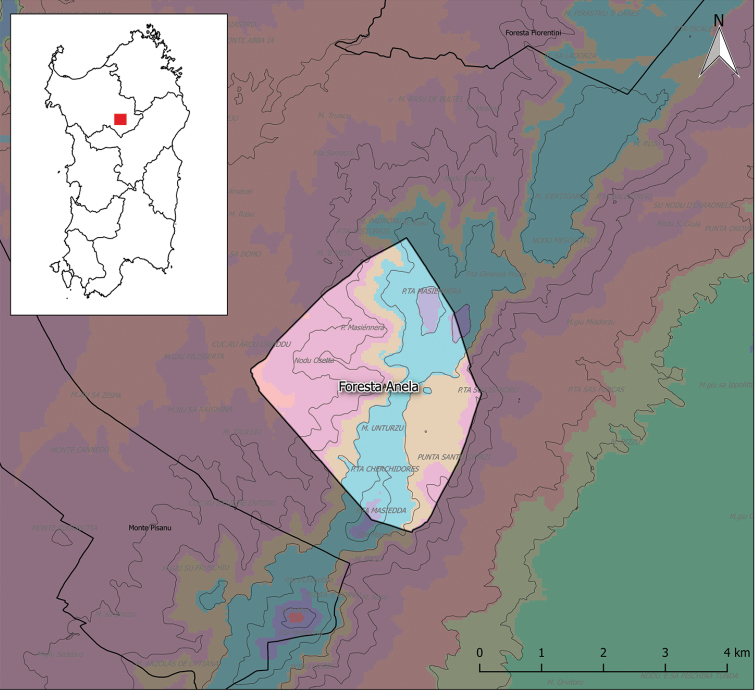
The study area, Forest of Anela and its location in Sardinia (red rectangle on the inset map). Colours on the map represent different isobioclimates (derived from Canu et al. 2015). In the domain, we can recognise five different isobioclimates: Violet: upper mesotemperate (subMediterranean), lower humid, weak semi-continental; blue: lower supraMediterranean, lower humid, weak semi-continental; orange: upper mesoMediterranean, lower humid, weak semi-continental; lilac: upper mesoMediterranean, upper subhumid, weak semi-continental; pink: upper mesoMediterranean, lower subhumid, weak semi-continental. Thick black lines represent domain limits; thin black lines represent altitude intervals of 100 m.

The forest domain of Anela is a public property since 1886, at present managed by the Sardinian regional agency Forestas (Fig. 1). The domain covers 1280 hectares of which 1200 ha fall in the municipality of Anela, 55 ha in that of Bultei (to the east) and 25 ha in that of Bono (to the west). The lowest altitude is about 600 m a.s.l. in locality *Badu Edras* whereas the summit point is at *Punta Masiedda* 1158 m a.s.l. The geographic coordinates of the forestry station headquarter are 40°27'14"N; 9°01'36"E. At present, the vegetation cover is mainly characterised by coppices and mature shrubs linked to sub-Mediterranean woods *Glechomosardoae*-*Quercetumcongestae* and *Saniculoeuropaeae*-*Quercetumilicis* above 800 m a.s.l. and meso-Mediterranean *Loncomelopyrenaici*-*Quercetumichnusae* and *Galioscabri*-*Quercetumilicis* below 800 m a.s.l., as described by Bacchetta et al. (2009). The 2004 forest census determined that 46% of this area was occupied by holm oak (*Quercusilex* L.) woods, 2.7% by deciduous oak (*Q.pubescens* Willd.) woods, 23.4% by mixed woods of holm oak and deciduous oak, 0.8% by cork oak (*Q.suber* L.) woods, 2.8% by plantations with alien trees (*Abies*, *Cedrus*, *Acer*, *Fagus*, *Pinus*), 14.7% by shrub communities (with *Ericaarborea*, *Crataegusmonogyna*, *Rubusulmifolius*), 6.2% by dwarf communities (with Helichrysummicrophyllumsubsp.tyrrhenicum, *Thymusherba-barona*, *Genistadesoleana*), 0.3% by rocky places and the rest by human activities (including buildings, an artificial lake and firebreaks) (Sechi and Falchi 2013). It should be noted that a large fire destroyed 800 hectares of the domain on 31 July 1945, so the wooded area decreased from 72.4% in 1910 to less than 20% in the 50s (Sechi and Falchi 2013).

In the ambit of the Sardinian-Corsican biogeographic province (as defined by Bacchetta et al. 2012), the study area falls in the Goceano-Logudorese sector (Fenu et al. 2014).

The geology of the study area comprises Palaeozoic granites and schists (Madrau 2013). The impermeable nature of these substrates has created a substantial aquifer evident by the presence of 39 springs (half perennial and half seasonal) in the study area (Farris 2013b).

Bioclimate analysis of 1971–2000 data (Canu et al. 2015) showed that 96.9% of the area falls in the Mediterranean Pluviseasonal Oceanic bioclimate, whereas 3.1% in the Temperate Oceanic bioclimate (submediterranean variant). A total of 64.6% of the area is included in the meso-Mediterranean thermotype, 32.3% in the supra-Mediterranean and 3.1% in the meso-Temperate.

Thermo-pluviometric data of the period 1951–1985 showed annual mean temperature of 11.2 °C and annual mean rainfall of 1040 mm; after the year 2000 temperatures did not vary significantly, whereas a reduction of ca. 30% in the annual rainfall was recorded. Late spring and summer rainfall (May-August) decreased even more (more than 50%, see Farris 2013a).

The study area is entirely included in the Natura 2000 site of community importance ITB 011102 ‘‘Catena del Marghine e Goceano’’, extended on 14,984 ha and is also nominated as a Protection Oasis for wildlife “Foresta Anela”, managed by the Province of Sassari.

### Floristic research

Floristic research started in the year 2000 and was intensified in the years 2012–17 with regular monthly sampling. Each month, we made one day excursions, which covered three altitudinal ranges (< 800 m a.s.l.; 800–1000 m a.s.l.; > 1000 m a.s.l. on the third). For each excursion, we tried to visit as many habitats as possible in order to capture the highest environmental heterogeneity. Collected plants were stored at the Herbarium SS, where we also searched for specimens collected in previous decades (if present, they are reported in the floristic list).

Plant names were derived from the Euro+Med PlantBase (Euro+Med 2006–2018), except for: a) families not already included in this database for which we referred to the Checklists of Italian Flora (Conti et al. 2005; Bartolucci et al. 2018), APG IV (APG 2016); b) the family Orchidaceae (for which we follow GIROS (2016)); c) the genus *Orobanche*, for which we follow Domina and Arrigoni (2007); d) the genus *Dianthus*, for which Bacchetta et al. (2010) is followed; e) and the species *Struthiopterisspicant* which we use in preference to *Blechnumspicant* (Gasper et al. 2016); f) for endemics, we also consulted Arrigoni et al. (1976–1991) and Peruzzi et al. (2014). The Italian floras (Pignatti 1982, 2017–2018) and the Sardinian flora (Arrigoni 2006–2015) were also consulted. When other relevant literature was followed, it is specified in the text.

Plant authorities and names were further verified using ‘The Plant List’, ‘The World Checklist of Selected Plant Families’ and ‘The International Plant Names Index’ (IPNI). Herbarium acronyms follow Thiers (2018).

The taxonomic circumscription of orders and families, as well as their sequence in the list was derived from Smith et al. (2006) for Pteridophytes; and APG III (APG 2009), APG IV (APG 2016) and Haston et al. (2009) for Angiosperms. Within each family, genera, species and subspecies are listed in alphabetical order. Species and subspecies are numbered progressively.

For each taxon we report:

Progressive number Scientific name (with authority) Biological type, Chorologic type

Abundance (locality(ies) of collection is(are) specified only for uncommon or range restricted taxa): Habitat

Notes (eventual)

Biological types are in accordance to Raunkiær (1934) and were verified on the collected samples and also in Pignatti (1982, 2017–2018); chorologic types were determined following maps reported in the Euro+Med PlantBase (Euro+Med 2006–2018) and again verified in Pignatti (1982, 2017–2018) and the other bibliographic sources reported in the text.

Geographical abbreviations are:

Atl. Atlantic;

Cauc. Caucasian;

Circumbor. circum-boreal;

Cosmop. cosmopolitan;

Endem. endemic;

Euras. Eurasian;

Eurimedit. euri-Mediterranean;

Europ. European;

Eurosib. Euro-Siberian;

It Italy;

Itc central Italy;

Its northern Italy;

Macaron. Macaronesian;

Medit. Mediterranean;

Medit. Mont. Mediterranean montane;

S. Europ. Orof. Southern European Orophylous;

Paleotemp. paleo-temperate;

Paleotrop. paleo-Tropical;

Sib. Siberian;

Stenomedit. Steno-Mediterranean;

Subatl. sub-Atlantic;

Subcosmop. sub-cosmopolitan;

Submedit. sub-Mediterranean;

Subtrop. sub-Tropical;

Turan. Turanian.

Here we consider as endemics *sensu stricto* all taxa limited to the Corsican-Sardinian biogeographic province (*sensu*Bacchetta et al. 2012), therefore including the Tuscan Archipelago. Other taxa are considered endemic *sensu lato*, which includes those present in western Mediterranean islands and continental areas – Calabria in Europe, Kabylies in Africa – as far as the Miocene part of the Hercynian chain (Hercynian endemics *sensu*Mansion et al. 2008). Finally, other endemics *sensu lato* are ‘administrative endemics’, i.e. taxa confined within Italian national borders (Peruzzi et al. 2014). For endemics, geographic abbreviations are as follows:

Ag Algeria;

AT Tuscan Archipelago;

Bl Balearic Islands;

Co Corsica;

Hy Hyères islands;

Sa Sardinia;

Si Sicily.

Abundance is expressed on the basis of the following criteria:

RR range restricted: taxa present in only one locality within the study area or covering a surface not exceeding 1 hectare, i.e. Mentharequieniisubsp.requienii;

U uncommon: taxa found in 2–5 localities within the study area, or covering a surface not exceeding 1 km^2^, i.e. *Arisarumvulgare*;

L localised: taxa present in 6 or more localities within the study area, but covering less than 2.5 km^2^, i.e. *Agrostiscapillaris*;

C common: taxa covering more than 2.5 km^2^, i.e. *Quercusilex*.

## Results

### Floristic list

#### Lycopodiopsida

**Isoetales**

Isoetaceae

1 *Isoeteshistrix* Bory G bulb, Stenomedit.-Atl.

U (Zuanne Cane Malu, near Mt. Masiennera): Temporary ponds

**Selaginellales**

Selaginellaceae

2 *Selaginelladenticulata* (L.) Spring Ch rept, Stenomedit.

C: Woods, wet cliffs

#### Polypodiopsida

**Osmundales**

Osmundaceae

3 *Osmundaregalis* L. G rhiz, Subcosmop.

L: *Alnusglutinosa* woods, streams

**Polypodiales**

Dennstaedtiaceae

4 Pteridiumaquilinum(L.)Kuhnsubsp.aquilinum G rhiz, Cosmop.

C: Woods, meadows, fringes, garrigues, shrublands

Pteridaceae

5 *Anogrammaleptophylla* (L.) Link T caesp, Cosmop.

L: Shady rocks and cliffs

Aspleniaceae

6 *Aspleniumadiantum-nigrum* L. H ros, Paleotemp.

C: Shady rocks and cliffs, sometimes woods

Notes: since the taxon has been excluded from the Sardinian flora by Marchetti (2004), Arrigoni (2006–2015) and Bartolucci et al. (2018), here we consider it as new for the Sardinian flora.

7 *Aspleniumonopteris* L. H ros, Subtrop.

C: Woods, sometimes cliffs

8 AspleniumceterachL.subsp.ceterach H ros, Euras.

L: Walls

9 *Aspleniumforeziense* Magnier H ros, NW-Medit.-Mont.

U (Badu Edras): Shady rocks and cliffs

Notes: the taxon has been excluded from the Sardinian flora by Marchetti (2004) and Bartolucci et al. (2018), but confirmed by Arrigoni (2006–2015).

10 AspleniumobovatumViv.subsp.obovatum H ros, Stenomedit.

U (Mt. Masiennera): Crevices at the top of the mountain

11 Aspleniumtrichomanessubsp.quadrivalens D.E. Mey. H ros, Cosmop.

C: Shady rocks and cliffs

Woodsiaceae

12 *Athyriumfilix-femina* (L.) Roth H Ros, Subcosmop.

L: Wet places, mainly *Alnusglutinosa* woods

Blechnaceae

13 *Struthiopterisspicant* (L.) F.W.Weiss H ros, Circumbor.

RR (Few individuals in a wet wood near Sos Sauccheddos spring): *Alnusglutinosa* wood

Dryopteridaceae

14 *Polystichumsetiferum* (Forssk.) Woyn. G rhiz, Circumbor.

C: Woods

Polypodiaceae

15 PolypodiumcambricumL.subsp.cambricum H ros, Eurimedit.

C: Rocks, big trees

16 *Polypodiuminterjectum* Shivas H ros, Paleotrop.

U (Bidighinzos): Shady rocks

#### Magnoliopsida

**Alismatales**

Araceae

17 *Arisarumvulgare* O. Targ. Tozz. G rhiz, Stenomedit.

U (Bonu Trau, Badde Cherchi, Badu Edras): Woods and shrubland (lower altutides)

18 ArumitalicumMill.subsp.italicum G rhiz, Stenomedit.

L: Fringes

19 *Arumpictum* L. f. G rhiz, Endem. Sa-Co-AT-Bl

RR (Su Pizzu Sa Pedra): at the base of a cliff

Notes: this taxon is not considered as an Italian endemic by Peruzzi et al. (2014)

20 *Lemnagibba* L. I nat, Subcosmop.

L: Wet places, standing water

21 *Lemnaminor* L. I nat, Subcosmop.

RR (Su Francallossu spring): standing water

**Dioscoreales**

Dioscoreaceae

22 *Dioscoreacommunis* (L.) Caddick & Wilkin G rad, Eurimedit.

C: Woods

**Liliales**

Colchicaceae

23 *Colchicumnanum* K. Perss. G bulb, Endem. Sa-Co

L: Wet pastures and meadows

Smilacaceae

24 *Smilaxaspera* L. NP, Subtrop.

C: Woods

Liliaceae

25 *Gageabohemica* (Zauschn.) Schult. & Schult.f. G bulb, Eurimedit.

C: Pastures

**Asparagales**

Orchidaceae

26 *Anacamptislaxiflora* (Lam.) R. M. Bateman, Pridgeon & M. W. Chase G bulb, Eurimedit.

L: Wet meadows

Specimen examined (syn. *Orchislaxiflora* Lam.): Funtana Arile, Anela, 08 June 1980, B. Corrias, S. Diana (SS)

27 *Anacamptislongicornu* (Poir.) R. M. Bateman, Pridgeon & M. W. Chase G bulb, W-Stenomedit.

Not found in the field during this research

Specimen examined (syn. *Orchislongicornu* Poir.): S’Isfundadu, Anela, 13 May 1965, B. Corrias (SS)

28 *Anacamptispapilionacea* (L.) R. M. Bateman, Pridgeon & M. W. Chase G bulb, Eurimedit.

C: Dry grasslands

Specimen examined (syn. *Orchispapilionacea* L.): Funtana Arile, Anela, 08 June 1980, B. Corrias, S. Diana (SS)

29 *Dactylorhizainsularis* (Sommier) Landwehr G bulb, W-Stenomedit.

Not found in the field during this research

Specimen examined (syn. *D.sambucina* (L.) Soó): S’Isfundadu, Anela, 13 May 1965, B. Corrias (SS)

30 *Limodorumabortivum* (L.) Sw. G rhiz, Eurimedit.

U (Littu Majore and Minda ‘e Bassu - Minda ‘e Supra): *Quercusilex* woods

31 *Orchisprovincialis* Balb. ex Lam. & DC. G bulb, Stenomedit.

L: Clearings, fringes

Specimens examined: S’Isfundadu, Anela, 13 May 1965, B. Corrias (2 specimens, SS)

32 *Serapiaslingua* L. G bulb, Stenomedit.

L: Wet meadows

Specimen examined: Funtana Arile, Anela, 08 June 1980, B. Corrias, S. Diana (SS)

33 *Spiranthesspiralis* (L.) Chevall. G rhiz, Europ.-Cauc.

U (Funtana Arile): Wet meadows

Iridaceae

34 *Crocusminimus* DC. G bulb, Endem. Sa-Co

C: Pastures

35 *Irispseudacorus* L. G rhiz, Euras.

U (Su Pranu): Flooded meadows, ponds

36 RomuleacolumnaeSebast. & Maurisubsp.columnae G bulb, Stenomedit.

C: Pastures

37 *Romulearequienii* Parl. G bulb, Endem. Sa-Co

C: Pastures

Asphodelaceae

38 AsphodelusramosusL.subsp.ramosus G rhiz, Stenomedit.

C: Perennial grasslands, pastures, garrigues

Amaryllidaceae

39 AlliumchamaemolyL.subsp.chamaemoly G bulb, W-Stenomedit.

L: Annual grasslands (lower altitudes)

40 Alliumguttatumsubsp.sardoum (Moris) Stearn G bulb, Stenomedit.

C: Pastures, meadows

41 *Alliumparciflorum* Viv. G bulb, Endem. Sa-Co

L: Garrigues, rocky habitats

42 *Alliumsubhirsutum* L. G bulb, W-Stenomedit.

C: Perennial grasslands

43 *Alliumtriquetrum* L. G bulb, W-Stenomedit.

C: Fringes, woods

44 *Alliumvineale* L. G bulb, Eurimedit.

L: Perennial grasslands

45 Leucojumaestivumsubsp.pulchellum (Salisb.) Briq. G bulb, Endem. Sa-Co-Bl

L: Wet meadows

Notes: This taxon is reported also in the Var (Southern France) (see: Tison and de Foucault 2014, Arrigoni 2006–2015; Pignatti 2017–2018) whereas the Euro+Med Plantbase considers it exclusive only in Sardinia, Corsica and the Balearic Islands.

46 *Pancratiumillyricum* L. G bulb, Endem. Sa-Co-AT

L: Garrigues

Asparagaceae

47 *Asparagusacutifolius* L. G rhiz, Stenomedit.

L: Woods and shrubland (lower altitudes)

48 *Drimiapancration* (Steinh.) J. C. Manning & Goldblatt G bulb, W-Stenomedit.

L: Grasslands

49 *Leopoldiacomosa* (L.) Parl. G bulb, Eurimedit.

C: Grasslands, pastures

50 *Ornithogalumcorsicum* Jord. & Fourr. G bulb, Endem. Sa-Co

C: Pastures

51 *Ornithogalumpyrenaicum* L. G bulb, Eurimedit.

C: Deciduous woods

52 *Prosperoautumnale* (L.) Speta G bulb, Eurimedit.

C: Grasslands, pastures

53 *Ruscusaculeatus* L. G rhiz, Eurimedit.

C: Woods

**Poales**

Typhaceae

54 *Typhaangustifolia* L. G rhiz, Circumbor.

L: Artificial lake, flooded areas, streams

Juncaceae

55 *Juncusarticulatus* L. G rhiz, Circumbor.

C: Wet meadows, temporary ponds

56 *Juncusbufonius* L. T caesp, Cosmop.

C: Temporary ponds, wet soils

57 *Juncuscapitatus* Weigel T scap, Medit.-Atl.

C: Temporary ponds

58 JuncuseffususL.subsp.effusus H caesp, Cosmop.

C: Wet meadows, temporary ponds

59 *Juncushybridus* Brot. T caesp, Medit.-Atl.

C: Temporary ponds

60 *Luzulaforsteri* (Sm.) DC. H caesp, Eurimedit.

C: Woods

Cyperaceae

61 *Carexcaryophyllea* Latourr. H scap, Euras.

C: Wet pastures and meadows

62 *Carexdistachya* Desf. H caesp, Stenomedit.

C: Woods

63 *Carexdivisa* Huds. G rhiz, Medit.-Atl.

C: Wet meadows and pastures, temporary ponds, ditches

64 *Carexdivulsa* Stockes H caesp, Eurimedit.

C: Fringes

65 *Carexmicrocarpa* Moris He, Endem. Sa-Co-AT-Itc

L: *Alnusglutinosa* woods, riparian vegetation

66 *Carexremota* L. H caesp, Europ.-Cauc.

U (Badu Addes): *Alnusglutinosa* wood

67 *Cyperuslongus* L. G rhiz, Paleotemp.

C: Wet meadows, riparian vegetation

Notes: some authors exclude the presence of this species from Sardinia (Desfayes 2004, Arrigoni 2006–2015, Bartolucci et al. 2018) and consider the presence of *Cyperusbadius* Desf. instead. In the Euro+Med Plantbase, *C.badius* is considered a heterotypic synonym of *C.longus*.

68 Eleocharispalustris(L.)Roem. & Schult.subsp.palustris G rhiz, Subcosmop.

L: Wet meadows

Gramineae (*nom. altr.*Poaceae)

69 *Aegilopsgeniculata* Roth T scap, Stenomedit.-Turan.

L: Annual grasslands

70 *Agrostiscapillaris* L. H caesp, Circumbor.

L: Wet pastures and meadows

Notes: this taxon is new for the Sardinian flora following Pignatti (1982), Conti et al. (2005), Arrigoni (2006–2015), Pignatti (2017–2018), Bartolucci et al. (2018) and the Euro+Med PlantBase.

71 AiracaryophylleaL.subsp.caryophyllea T scap, Subtrop.

C: Annual grasslands

72 AlopecurusbulbosusGouansubsp.bulbosus H caesp, Eurimedit.-Subatl.

L: Wet pastures and meadows

73 *Anisanthadiandra* (Roth) Tutin T scap, Eurimedit.

C: Annual grasslands

74 Anisanthamadritensis(L.)Nevskisubsp.madritensis T scap, Eurimedit.

C: Annual grasslands, pastures

75 *Anthoxanthumodoratum* L. H caesp, Euras.

C: Wet pastures and meadows

76 Arrhenatherumelatiussubsp.sardoum (Em. Schmid) Gamisans H caesp, W-Stenomedit.

L: Garrigues, rocky habitats (higher altitudes)

77 AvenabarbataLinksubsp.barbata T scap, Eurimedit.

C: Annual grasslands

78 *Brachypodiumretusum* (Pers.) P. Beauv. H caesp, W-Stenomedit.

C: Perennial grasslands on rocky or stony soils

79 Brachypodiumsylvaticum(Huds.)P. Beauv.subsp.sylvaticum H caesp, Paleotemp.

C: Woods, fringes

80 *Brizamaxima* L. T scap, Subtrop.

C: Annual grasslands, pastures

81 *Brizaminor* L. T scap, Subcosmop.

U (near Mt. Masiennera): Wet pastures and meadows

82 BromushordeaceusL.subsp.hordeaceus T scap, Subcosmop.

C: Annual grasslands, pastures

83 *Bromusscoparius* L. T scap, Stenomedit.

U (Top of Mt. Masiennera): Annual grasslands

84 *Catabrosaaquatica* (L.) P. Beauv. G rhiz, Circumbor.

L: Wet soils

85 *Cynodondactylon* (L.) Pers. G rhiz, Cosmop.

C: Wet pastures and meadows

86 *Cynosuruscristatus* L. H caesp, Europ.-Cauc.

C: Wet pastures and meadows

87 *Cynosurusechinatus* L. T scap, Eurimedit.

C: Annual grasslands, fringes

88 *Cynosuruseffusus* Link T scap, Stenomedit.

C: Annual grasslands, fringes

89 Dactylisglomeratasubsp.hispanica (Roth) Nyman H caesp, Stenomedit.

C: Perennial grasslands

90 Danthoniadecumbens(L.)DC.subsp.decumbens H caesp, Europ.

L: Wet pastures and meadows

91 *Dasypyrumvillosum* (L.) P. Candargy T Scap, Eurimedit.-Turan.

L: Annual grasslands

92 FestucamorisianaParl.subsp.morisiana H caesp, Endem. Sa

L: Wet meadows and pastures

93 *Glycerianotata* Chevall. G rhiz, Subcosmop.

L: Wet habitats

94 HolcuslanatusL.subsp.lanatus H caesp, Circumbor.

C: Wet meadows

95 *Hordeumgeniculatum* All. T scap, Stenomedit.

C: Wet meadows and pastures, temporary ponds

96 LagurusovatusL.subsp.ovatus T scap, Eurimedit.

C: Annual grasslands, pastures

97 LoliumperenneL.subsp.perenne H caesp, Euras.

C: Wet pastures

98 LoliumrigidumGaudinsubsp.rigidum T scap, Subtrop.

C: Pastures on arid soil

99 MelicaciliataL.subsp.ciliata H caesp, Eurimedit.

U (Mt. Masiennera): Rocky habitats

100 *Melicaminuta* L. H caesp, Stenomedit.

C: Fringes

101 *Melicauniflora* Retz. H caesp, Paleotemp.

L: Deciduous woods, fringes

102 *Neoschischkiniapourrettii* (Willd.) Valdés & H. Scholz T scap, W-Stenomedit.

L: Temporary ponds

103 Piptatherummiliaceum(L.)Coss.subsp.miliaceum H caesp, Stenomedit.

L: Road edges (lower altitudes)

104 PoaannuaL.subsp.annua T caesp, Cosmop.

C: Annual grasslands, pastures

105 *Poabalbisii* Parl. H caesp, Endem. Sa-Co

U (Mt. Masiennera): Garrigues, rocky habitats

106 PoabulbosaL.subsp.bulbosa H caesp, Paleotemp.

C: Pastures

107 *Poainfirma* Kunth T caesp, Eurimedit.

C: Mud, wet soils

108 PoanemoralisL.subsp.nemoralis H caesp, Circumbor.

C: Woods

109 PoatrivialisL.subsp.trivialis H caesp, Euras.

C: Wet meadows

110 *Vulpialigustica* (All.) Link T caesp, Stenomedit.

C: Pastures

111 Vulpiamyuros(L.)C. C. Gmel.subsp.myuros T caesp, Subcosmop.

C: Pastures

112 *Vulpiasicula* (C. Presl) Link H caesp, W-Medit.-Mont.

C: Pastures, grasslands

**Ranunculales**

Papaveraceae

113 *Fumariabastardii* Boreau T scap, Subatl.

C: Annual grasslands, fringes

114 FumariaofficinalisL.subsp.officinalis T scap, Paleotemp.

C: Annual grasslands, fringes

115 PapaverrhoeasL.subsp.rhoeas T scap, E-Medit.

C: Pastures, grasslands

Ranunculaceae

116 AnemonehortensisL.subsp.hortensis G bulb, N-Medit.

RR (Su Tattharesu): Perennial grasslands

117 *Clematisvitalba* L. P lian, Europ.-Cauc.

C: Woods, mantles

118 FicariavernaHuds.subsp.verna. G bulb, Euras.

C: Woods

119 Ranunculusbulbosussubsp.aleae (Willk.) Rouy & Foucaud H scap, Euras.

C: Grasslands, fringes, woods

120 RanunculusbullatusL.subsp.bullatus H ros, Stenomedit.

C: Annual grasslands

121 RanunculuscordigerViv.subsp.cordiger H scap, Endem. Sa-Co

L: Wet meadows, temporary ponds

122 *Ranunculusmacrophyllus* Desf. H scap, SW-Medit.

L: Wet meadows

123 *Ranunculusmuricatus* L. T scap, Eurimedit.

C: Mud, wet meadows

124 *Ranunculusophioglossifolius* Vill. T scap, Eurimedit.

L: Mud, temporary ponds

125 RanunculuspaludosusPoir.subsp.paludosus H scap, Stenomedit.

C: Pastures

126 *Ranunculussardous* Crantz T scap, Eurimedit.

C: Mud, temporary ponds

**Saxifragales**

Paeoniaceae

127 *Paeoniacorsica* Tausch G rhiz, Endem. Sa-Co

L: Woods, clearings

Saxifragaceae

128 *Saxifragatridactylites* L. T scap, Eurimedit.

L: Annual grasslands

Crassulaceae

129 *Sedumcaeruleum* L. T scap, SW-Medit.

C: Rocky habitats, annual grasslands

130 *Sedumcepaea* L. T scap, Submedit.-Subatl.

C: Rocky habitats, annual grasslands

131 *Sedumrubens* L. T scap, Eurimedit.-Subatl.

C: Rocky habitats, annual grasslands

132 *Sedumstellatum* L. T scap, Stenomedit.

C: Rocky habitats, annual grasslands

133 Sedumvillosumsubsp.glandulosum (Moris) P. Fourn. H scap, Endem. Sa-Ag

C: Rocky habitats, annual grasslands

134 *Umbilicusrupestris* (Salisb.) Dandy subsp. rupestris G bulb, Medit.-Atl.

C: Rocky habitats

**Fabales**

Leguminosae (*nom. altr.*Fabaceae)

135 *Cytisusvillosus* Pourr. P caesp, W-Stenomedit.

C: Shrubland, mantles

136 *Dorycniumrectum* (L.) Ser. H scap, Stenomedit.

L: Wet habitats

137 *Genistacorsica* (Loisel.) DC. NP, Endem. Sa-Co

L: Garrigues on rocky soils

138 *Genistadesoleana* Vals. NP, Endem. Sa-Co-Its

C: Garrigues, dwarf shrubs

Specimens examined: Punta Chelchidores, Anela, 18 July 1972, F. Valsecchi (3 specimens, SS)

139 *Lathyrusaphaca* L. T scap, Eurimedit.

C: Pastures, fringes

140 *Lathyrussphaericus* Retz. T Scap, Eurimedit.

L: Pastures

141 *Lotusalpinus* (DC.) Ramond H scap, Orof. S-Europ.

C: Wet pastures and meadows

142 *Lotusangustissimus* L. T scap, Eurimedit.

L: Temporary ponds

143 *Lotusconimbricensis* Brot. T scap, W- Stenomedit.

C: Annual grasslands

144 *Lotushispidus* DC. T scap, W-Medit.

C: Annual grasslands

145 LupinusangustifoliusL.subsp.angustifolius T scap, Stenomedit.

C: Annual grasslands

146 *Medicagopolymorpha* L. T scap, Eurimedit.

C: Pastures, annual grasslands

147 OnonisspinosaL.subsp.spinosa Ch suffr, Eurimedit.

C: Grasslands, pastures

148 *Ornithopuscompressus* L. T scap, Eurimedit.

C: Annual grasslands

149 *Ornithopuspinnatus* (Mill.) Druce T Scap, Medit.-Atl.

L: Pastures

150 *Trifoliumangustifolium* L. T scap, Eurimedit.

C: Annual grasslands

151 *Trifoliumarvense* L. T scap, Paleotemp.

C: Pastures

152 *Trifoliumcampestre* Schreb. T scap, Paleotemp.

C: Annual grasslands

153 *Trifoliumglomeratum* L. T Scap, Eurimedit.

L: Pastures

154 Trifoliumincarnatumsubsp.molinerii (Hornem.) Syme T scap, Eurimedit.

C: Grasslands, pastures

155 *Trifoliummicranthum* Viv. T scap, Paleotemp.

C: Annual grasslands

156 TrifoliumnigrescensViv.subsp.nigrescens T scap, N-Medit.

C: Pastures

157 *Trifoliumpratense* L. H scap, Eurosib.

C: Wet meadows and pastures

158 Trifoliumrepenssubsp.prostratum Nyman H rept, Eurimedit.

C: Wet meadows and pastures

159 *Trifoliumspumosum* L. T scap, Stenomedit.

C: Annual grasslands

160 *Trifoliumsquarrosum* L. T scap, Eurimedit.

L: Pastures

161 *Trifoliumstellatum* L. T scap, Eurimedit.

C: Annual grasslands, pastures

162 Trifoliumsubterraneumsubsp.yanninicum Katzn. & F. H. W. Morley T rept, E-Medit.

C: Pastures

163 *Trifoliumtomentosum* L. T rept, Paleotemp.

C: Annual grasslands, pastures

164 ViciacraccaL.subsp.cracca H scap, Euras.

C: Fringes

165 *Vicialathyroides* L. T scap, Eurimedit.

C: Fringes

166 VicialuteaL.subsp.lutea T scap, Eurimedit.

C: Fringes

167 Viciavillosasubsp.ambigua (Guss.) Kerguélen H Scap, W-Stenomedit.

L: Fringes

168 ViciavillosaRothsubsp.villosa T scap, Eurimedit.

C: Fringes

**Rosales**

Rosaceae

169 AgrimoniaeupatoriaL.subsp.eupatoria H scap, Subcosmop.

C: Fringes

170 *Crataegusmonogyna* Jacq. P caesp, Paleotemp.

C: Shrublands, woods, mantles

171 FragariavescaL.subsp.vesca H rept, Eurosib.

C: Deciduous woods, fringes

172 *Geumurbanum* L. H scap, Circumbor.

C: Deciduous woods, fringes

Specimen examined: Caserma Forestale Anela, sine die, Barba (SS)

173 *Maluspumila* Mill. P scap, CW-Euras.

L: Woods, mantles

Notes: in accordance with Bagella and Urbani (2006), this is the valid name for *Malusdomestica* Borkh. (nom. illeg.), also reported in the Euro+Med PlantBase. Yet Galasso et al. (2018) call a taxon *Malusdomestica*, considering it as a non-native species, while Camarda and Valsecchi (2008), Arrigoni (2006–2015) and Pignatti (2017–2018) still call it *M.dasyphylla*. Finally, Bartolucci et al. (2018) report the taxon *M.sylvestris* in Sardinia. *Maluspumila* is reported as a synonym of *M.domestica* by Galasso et al. (2018), it is excluded from the Sardinian flora by Arrigoni (2006–2015), finally, it was not mentioned by Camarda and Valsecchi (2008). In the Euro+Med Plantbase, *Maluspumila* Mill. is the valid name for *Malusdomestica* Borkh. The populations we have examined in the Marghine-Goceano range (not only the forest domain of Anela) have the characters of *Malusdomestica*, not *M.sylvestris*.

174 *Potentillareptans* L. H ros, Paleotemp.

C: Wet meadows

175 *Prunusavium* (L.) L. P scap, Pontic

L: Woods

176 Prunusdomesticasubsp.insititia (L.) Bonnier & Layens P scap

U (Su Cantareddu): Mantles

177 PrunusspinosaL.subsp.spinosa P caesp, Europ.-Cauc.

C: Shrublands

178 Pyruscommunissubsp.pyraster (L.) Ehrh. P scap, Euras.

L: Woods, mantles

179 *Pyrusspinosa* Forssk. P caesp, Stenomedit.

C: Shrublands, mantles, woods

180 *Rosacanina* L. NP, Paleotemp.

C: Shrublands

181 *Rosasempervirens* L. NP, Stenomedit.

L: Woods, shrublands (lower altitudes)

182 *Rosasubcanina* (Christ) Vuk. NP, Europ.

C: Shrublands

183 *Rubusulmifolius* Schott NP, Eurimedit.

C: Shrublands, woods

184 Sanguisorbaminorsubsp.balearica (Bourg. ex Nyman) Muñoz Garm. & C. Navarro H scap, Eurimedit.

C: Grasslands

Ulmaceae

185 UlmusminorMill.subsp.minor P caesp, Europ.-Cauc.

L: Woods

Cannabaceae

186 CeltisaustralisL.subsp.australis P scap, Eurimedit.

RR (Pedru Addes): Wood edge

Moraceae

187 FicuscaricaL.subsp.carica P scap, Medit.-Turan.

U (Badu Edras): Riparian vegetation

Urticaceae

188 ParietarialusitanicaL.subsp.lusitanica T rept, Stenomedit.

C: Buildings, fringes

189 *Urticaatrovirens* Loisel. H scap, Endem. Sa-Co-Bl-AT-Itc

L: Ruderal vegetation

190 UrticadioicaL.subsp.dioica H scap, Subcosmop.

C: Ruderal vegetation

**Fagales**

Fagaceae

191 *Quercusilex* L. P scap, Stenomedit.

C: Woods

192 *Quercuspubescens* Willd. agg. P caesp, SE-Europ.

C: Woods

Notes: There are many controversial treatments for describing the variation within *Q.pubescens* (Mossa et al. 1998, 1999). Until the various treatments are resolved, we prefer to treat this variation as a complex (or aggregate) within *Q.pubescens* s.l.

193 *Quercussuber* L. P scap, W-Eurimedit.

L: Woods

Betulaceae

194 Alnusglutinosa(L.)Gaertn.subsp.glutinosa P scap, Paleotemp.

L: Streams, wet places, springs

**Oxalidales**

Oxalidaceae

195 OxaliscorniculataL.subsp.corniculata H rept, Eurimedit.

L: Walls, buildings

**Malpighiales**

Guttiferae (*nom. altr.*Clusiaceae)

196 *Hypericumandrosaemum* L. NP, W-Eurimedit.-Subatl.

L: Wet habitats, springs

197 HypericumhircinumL.subsp.hircinum NP, Endem. Sa-Co-AT

L: Springs, streams, *Alnusglutinosa* woods

Notes: *H.hircinum* includes several subspecies, amongst which the subsp. hircinum is exclusive of Sardinia, Corsica and the Tuscan Archipelago (Carta and Peruzzi 2015)

198 HypericumperforatumL.subsp.perforatum H scap, Paleotemp.

C: Fringes, road edges

Violaceae

199 Violaalbasubsp.dehnhardtii (Ten.) W. Becker H ros, Eurimedit.

C: Woods, fringes

200 *Violareichenbachiana* Jord. ex Boreau H scap, Eurosib.

C: Deciduous woods

Notes: it was excluded for the Sardinian flora by Arrigoni (2006–2015), but later confirmed by Mereu (2012) for the Gennargentu massif

Salicaceae

201 Salixcinereasubsp.oleifolia Macreight P caesp, W-Medit.-Atl.

L: Streams, springs

202 *Salixpurpurea* L. P scap, Euras.

L: Ditches

Euphorbiaceae

203 EuphorbiacharaciasL.subsp.characias NP, Stenomedit.

C: Woods, shrublands (lower altitudes)

204 EuphorbiahelioscopiaL.subsp.helioscopia T scap, Cosmop.

C: Annual grasslands

205 Euphorbiapithyusasubsp.cupanii (Guss. ex Bertol.) Radcl.-Sm. G rhiz, Endem. Sa-Co-Si

C: Perennial grasslands, pastures

206 *Euphorbiasemiperfoliata* Viv. G rhiz, Endem. Sa-Co

L: Woods, fringes

Linaceae

207 *Linumbienne* Mill. H bienn, Eurimedit.

C: Annual grasslands

**Geraniales**

Geraniaceae

208 *Erodiumchium* (L.) Willd. T scap, Eurimedit.

L: Pastures

209 *Erodiumciconium* (L.) L’Hér. T scap, Eurimedit.-Pontic

C: Pastures

210 *Erodiumcicutarium* (L.) L’Hér. T scap, Subcosmop.

C: Pastures

211 *Geraniumpurpureum* Vill. T scap, Eurimedit.

C: Woods, fringes

212 *Geraniumrobertianum* L. T scap, Subcosmop.

C: Woods, fringes

213 *Geraniumrotundifolium* L. T scap, Paleotemp.

C: Woods, fringes

**Myrtales**

Lythraceae

214 *Lythrumportula* (L.) D. A. Webb T rept, S-Europ.-S-Sib.

L: Temporary ponds

Onagraceae

215 *Epilobiummontanum* L. H scap, Euras.

C: Woods

**Sapindales**

Sapindaceae

216 AcermonspessulanumL.subsp.monspessulanum P caesp, Eurimedit.

L: Woods and mantles

**Malvales**

Malvaceae

217 *Althaeahirsuta* L. T scap, Eurimedit.

L: Annual grasslands

218 *Malvaolbia* (L.) Alef. P caesp, Stenomedit.

C: Shrublands on wet soils

219 *Malvasylvestris* L. H scap, Eurosib.

C: Grasslands, fringes

Cistaceae

220 *Cistusmonspeliensis* L. NP, Stenomedit.

C: Garrigues (lower altitudes)

221 *Cistussalviifolius* L. NP, Stenomedit.

C: Garrigues

222 *Tuberariaguttata* (L.) Fourr. T scap, Eurimedit.

C: Annual grasslands

**Brassicales**

Resedaceae

223 Sesamoidespurpurascenssubsp.spathulata (Moris) Lambinon & Kerguélen H Scap, W-Medit.-Mont.

C: Dirty tracks, trampled places

Cruciferae (*nom. altr.*Brassicaceae)

224 *Arabidopsisthaliana* (L.) Heynh. T scap, Paleotemp.

C: Annual grasslands, pastures

225 Capsellabursapastoris(L.)Medik.subsp.bursa-pastoris H bienn, Cosmop.

C: Annual grasslands, pastures

226 *Cardamineflexuosa* With. H scap, Circumbor.

C: Fringes

227 *Cardaminehirsuta* L. T scap, Cosmop.

C: Fringes

228 *Drabamuralis* L. T scap, Circumbor.

L: Cliffs, road edges

229 Erophilavernasubsp.praecox (Steven) Walters T scap, Eurimedit.

C: Annual grasslands

230 *Morisiamonanthos* (Viv.) Asch. H ros, Endem. Sa-Co

U (Near Mt. Masiennera): Wet meadows

231 *Nasturtiumofficinale* (L.) R. Br. H scap, Cosmop.

L: Muds, streams

232 RaphanusraphanistrumL.subsp.raphanistrum T scap, Eurimedit.

C: Grasslands

233 *Sisymbriumofficinale* (L.) Scop. T scap, Paleotemp.

C: Pastures

234 *Teesdaliacoronopifolia* (J.P. Bergeret) Thell. T scap, Eurimedit.

C: Pastures

**Santalales**

Santalaceae

235 *Osyrisalba* L. NP, Eurimedit.

L: Woods, clearings, rocky habitats

**Caryophyllales**

Plumbaginaceae

236 ArmeriasardoaSpreng.subsp.sardoa Ch suffr, Endem. Sa

L: Garrigues, rocky habitats

Polygonaceae

237 RumexbucephalophorusL.subsp.bucephalophorus T scap, Eurimedit.-Macaron.

C: Annual grasslands

238 *Rumexcrispus* L. H scap, Subcosmop.

C: Wet meadows

239 RumexpulcherL.subsp.pulcher H scap, Eurimedit.

C: Wet meadows

240 Rumexscutatussubsp.glaucescens (Guss.) Brullo, Scelsi & Spamp. H scap, Endem. Sa-Si

L: Rocky habitats

241 *Rumexthyrsoides* Desf. H scap, W-Medit.

C: Fringes

Caryophyllaceae

242 *Arenariabalearica* L. Ch suffr, Endem. Sa-Co-Bl-AT

L: Shady rocks and cliffs

Specimens examined: S’Isfundadu, Anela, 25 May 1966, B. Corrias (2 specimens, SS); S’Isfundadu, Anela, 18 June 1965, F. Valsecchi (1 specimen, SS).

243 *Cerastiumgibraltaricum* Boiss. Ch suffr, Orof. W-Medit.

L: Garrigues

Notes: in the Euro+Med Plantbase, *Cerastiumboissierianum* Greuter et Burdet is considered a synonym of *C.gibraltaricum*

244 *Cerastiumglomeratum* Thuill. T scap, Eurimedit.

C: Pastures

245 Cerastiumligusticumsubsp.palustre (Moris) P. D. Sell et Whitehead T scap, Endem. Sa-Co

RR (near Mt. Masiennera): Wet pastures and meadows

246 *Corrigiolatelephiifolia* Pourr. H Ros, W-Medit.

L: Trampled sites, dirty roads

Specimen examined: Badu Addes, Anela, September 1962 (sine die), sine coll. (SS)

247 Dianthusichnusaesubsp.toddei Bacch., Brullo, Casti et Giusso H scap, Endem. Sa

L: Garrigues, rocky habitats

Notes: this taxon is exclusive for the Goceano mountain range (Bacchetta et al. 2010).

248 *Moenchiaerecta* (L.) P. Gaertn., B. Mey. & Scherb. subsp. erecta T scap, Medit.-Atl.

C: Pastures

249 *Petrorhagiadubia* (Raf.) G. López & Romo T scap, S-Medit.

C: Pastures

250 *Petrorhagiasaxifraga* (L.) Link H caesp, Eurimedit.

C: Garrigues, rocky habitats

251 *Saginaapetala* Ard. T scap, Eurimedit.

L: Annual grasslands, dirty tracks

252 *Saginaprocumbens* L. H caesp, Subcosmop.

L: Wet places, springs

Specimen seen: Badu Addes, Anela, sine die, Barba (SS)

253 *Saginasubulata* (Sw.) C. Presl H caesp, Medit.-Atl.

L: Wet meadows, rocky habitats (higher altitudes)

Notes: for this taxon, recently the name *S.alexandrae* Iamonico has been proposed (Iamonico 2016)

254 *Silenegallica* L. T scap, Eurimedit.

C: Pastures

255 *Silenelaeta* (Aiton) Godr. T scap, W-Stenomedit.

L: Muddy places, wet meadows, temporary ponds

256 *Silenelatifolia* Poir. H bienn, Paleotemp.

C: Fringes

257 Silenevulgaris(Moench)Garckesubsp.vulgaris H scap, Paleotemp.

C: Fringes

258 *Spergulaarvensis* L. T scap, Subcosmop.

C: Pastures

259 Stellariamedia(L.)Cirillosubsp.media T rept, Cosmop.

C: Ruderal vegetation, woods, fringes

Amaranthaceae

260 ChenopodiumalbumL.subsp.album T Scap, Subcosmop.

Not found in the field during this research

Specimens examined: Badu Addes, Anela, 09 September 1962, Barba (2 specimens, SS).

Portulacaceae

261 Montiafontanasubsp.amporitana Sennen T scap, Medit-Mont. Subatl.

C: Mud, flooded soils

**Ericales**

Primulaceae

262 *Anagallisarvensis* L. T rept, Eurimedit.

C: Annual grasslands

263 *Asterolinonlinum-stellatum* (L.) Duby T Scap, Stenomedit.

L: Annual grasslands, pastures

264 CyclamenrepandumSibth. & Sm.subsp.repandum G bulb, NW-Stenomedit.

C: Woods

Ericaceae

265 *Arbutusunedo* L. P caesp, Stenomedit.

RR (Littu Majore): Wood

266 *Ericaarborea* L. P caesp, Stenomedit.

C: Shrublands, woods

**Gentianales**

Rubiaceae

267 *Cruciataglabra* (L.) Ehrend. H scap, Euras.

C: Grasslands, pastures

Specimen examined: Badu Addes, Anela, 18 July 1972, B. Corrias, S. Diana, F. Valsecchi (SS).

268 GaliumaparineL.subsp.aparine T scap, Euras.

C: Fringes

269 *Galiumcorsicum* Spreng. H scap, Endem. Sa-Co

L: Rocky habitats

270 *Galiumdebile* Desv. H scap, Eurimedit.

L: Wet habitats

271 *Galiumrotundifolium* L. H scap, Orof.-W-Euras.

L: Woods (higher altitudes)

272 RubiaperegrinaL.subsp.peregrina P lian, Stenomedit.-Macaron.

C: Woods

273 *Sherardiaarvensis* L. T scap, Eurimedit.

C: Pastures, annual grasslands

274 *Theligonumcynocrambe* L. T scap, Stenomedit.

C: Annual grasslands, fringes

Gentianaceae

275 *Exaculumpusillum* (Lam.) Caruel T scap, W-Eurimedit.

RR (Minda ‘e Bassu): Temporary pond

**Boraginales**

Boraginaceae

276 *Anchusahybrida* Ten. H scap, Stenomedit.

Not found in the field during this research

Specimens examined: Badu Addes, Anela, 22 October 1963, F. Valsecchi, Barba (3 specimens, SS).

277 *Cynoglossumcreticum* Mill. H bienn, Eurimedit.

L: Fringes

278 *Echiumplantagineum* L. T Scap, Eurimedit.

C: Pastures, grasslands

279 Myosotisarvensis(L.)Hillsubsp.arvensis T scap, Europ.-W-Asian

C: Annual grasslands, pastures

280 *Myosotissicula* Guss. T scap, N-Eurimedit.

L: Wet meadows, temporary ponds

Convolvulaceae

281 *Convolvulusalthaeoides* L. H scand, Stenomedit.

C: Perennial grasslands

282 *Convolvulusarvensis* L. G rhiz, Paleotemp.

C: Perennial grasslands

283 Cuscutaepithymumsubsp.corsicana (Yunck.) Lambinon T par, Endem. Sa-Co

L: Garrigues (mainly parasite on *Genistadesoleana*)

**Solanales**

Solanaceae

284 *Solanumdulcamara* L. NP, Paleotemp.

U (Su Pranu): Riparian vegetation

**Lamiales**

Oleaceae

285 *Phillyrealatifolia* L. P caesp, Stenomedit.

C: Woods, shrubland (lower altitude)

Plantaginaceae

286 *Callitrichestagnalis* Scop. I rad, Euras.

L: Temporary ponds, springs, muddy soils

287 Cymbalariaaequitriloba(Viv.)A. Chev.subsp.aequitriloba Ch rept, Endem. Sa-Co-Bl-AT

L: Shady rocks and cliffs

288 DigitalispurpureaL.subsp.purpurea H scap, W-Eurimedit.

C: Fringes, clearings

289 *Linariaarvensis* (L.) Desf. T scap, Submedit.-Subatl.

C: Annual grasslands

290 *Linariapelisseriana* (L.) Mill. T scap, Medit.-Atl.

C: Pastures

291 *Plantagocoronopus* L. T scap, Eurimedit.

C: Grasslands, pastures

292 PlantagolagopusL.subsp.lagopus T scap, Stenomedit.

C: Annual grasslands, pastures

293 *Plantagolanceolata* L. H ros, Euras.

C: Grasslands

294 PlantagomajorL.subsp.major H ros, Euras.

L: Wet meadows

295 *Plantagoweldenii* Rchb. T scap, Stenomedit.

C: Annual grasslands

296 Veronicaanagallis-aquaticaL.subsp.anagallis-aquatica H scap, Cosmop.

L: Mud, springs, ditches

Specimen examined: Punta Chelchidores est, Anela, 18 July 1972, B. Corrias, S. Diana, F. Valsecchi (SS)

297 *Veronicaarvensis* L. T scap, Subcosmop.

C: nitrophilous vegetation

298 VeronicahederifoliaL.subsp.hederifolia T scap, Euras.

C: Woods, fringes

299 Veronicavernasubsp.brevistyla (Moris) Rouy T scap, Endem. Sa-Co

L: Pastures (higher altitudes)

Scrophulariaceae

300 *Scrophulariatrifoliata* L. H caesp, Endem. Sa-Co-AT

L: Rocky habitats

Specimen examined: Badu Addes, Anela, 18 July 1972, F. Valsecchi (SS)

301 ScrophulariaumbrosaDumort.subsp.umbrosa H Scap, Euras.

Not found in the field during this research

Specimens examined: Badu Addes, Anela, 18 July 1973, F. Valsecchi (3 specimens, SS)

302 *Verbascumpulverulentum* Vill. H bienn, Europ.

C: Clearings, fringes

Labiatae (*nom. altr.*Lamiaceae)

303 Clinopodiumnepetasubsp.glandulosum (Req.) Govaert H scap, Stenomedit.

C: Fringes

304 Clinopodiumvulgaresubsp.orientale Bothmer H scap, E-Stenomedit.

C: Fringes

Notes: The Italian Flora Checklists (Conti et al. 2005, Bartolucci et al. 2018) consider the subsp. arundanum (Boiss.) Nyman as present in Sardinia, whereas, the Euro+Med PlantBase considers subsp. arundanum absent from the island (and the whole Italian peninsula) and that, instead, subsp. orientale is present. Our specimens fit well with the diagnostic characters of subsp. orientale as described by Bothmer (1967).

305 *Glechomasardoa* (Bég.) Bég. H rept, Endem. Sa

L: Woods, fringes

306 *Lamiummaculatum* (L.) L. H scap, Euras.

U: Forest near forestry headquarters, under *Quercusilex*

Notes: according to Arrigoni (2006–2015), this taxon was not found in Sardinia in recent years

307 *Lamiumpurpureum* L. T scap, Euras.

C: Fringes

308 LavandulastoechasL.subsp.stoechas NP, Stenomedit.

C: Garrigues

309 *Menthaaquatica* L. H scap, Paleotemp.

L: Wet meadows

310 MenthapulegiumL.subsp.pulegium H scap, Eurimedit.

C: Wet meadows, temporary ponds

311 MentharequieniiBenth.subsp.requienii H rept, Endem. Sa-Co

RR (Su Cantareddu spring): Wet rocks, spring

312 Menthasuaveolenssubsp.insularis (Req. ex Gren. & Godr.) Greuter H scap, Endem. Sa-Co-AT-Bl

U (Funtana Arile spring): Fringes

313 Micromeriagraeca(L.)Benth.subsp.graeca Ch suffr, Stenomedit.

C: Garrigues

314 PrunellavulgarisL.subsp.vulgaris H scap, Circumbor.

C: Wet meadows, fringes, clearings

315 *Salviaverbenaca* L. H scap, Medit.-Atl.

C: Grasslands

Notes: following the Euro+Med PlantBase, in this taxon we include ecotypes referred to *Salviaclandestina* L.

316 *Stachysarvensis* (L.) L. T scap, Europ.

L: Annual grasslands, pastures

317 *Stachyscorsica* Pers. H rept, Endem. Sa-Co

L: Shady rocks and cliffs

Specimens examined: S’Isfundadu, Anela, 18 June 1965, F. Valsecchi (SS); Badu Addes, Anela, 18 July 1972, B. Corrias, S. Diana, F. Valsecchi (SS)

318 *Stachysglutinosa* L. Ch frut, Endem. Sa-Co-AT

L: Garrigues, rocky habitats

319 TeucriumchamaedrysL.subsp.chamaedrys Ch suffr, Eurimedit.

U (near the helicopter base): Pastures, grasslands

320 *Thymusherba-barona* Loisel. Ch rept, Endem. Sa-Co-Bl

C: Garrigues

Orobanchaceae

321 *Orobanchehederae* Duby T par, Eurimedit.

C: Woods

322 *Orobancheminor* Sm. T par, Paleotemp.

C: Grasslands, pastures

323 *Orobanchenana* (Reut.) Beck T par, Medit.-Macaron.

L: Grasslands, pastures

324 *Orobancheramosa* L. T par, Paleotemp.

L: Road sides, pastures

325 *Orobancherapum-genistae* Thuill. T par, Subatl.

L: Garrigues with *Genista* sp.

326 *Orobancherigens* Loisel. T par, Endem. Sa-Co

L: Garrigues with *Genista* sp.

327 Parentucellialatifolia(L.)Caruelsubsp.latifolia T scap, Eurimedit.

C: Pastures

328 *Parentucelliaviscosa* (L.) Caruel T scap, Medit.-Atl.

C: Annual grasslands

**Aquifoliales**

Aquifoliaceae

329 *Ilexaquifolium* L. P caesp, Submedit.-Subatl.

C: Woods

**Asterales**

Campanulaceae

330 *Jasionemontana* L. H scap, Europ.-Cauc.

C: Pastures and rocky habitats

Compositae (*nom. altr.*Asteraceae)

331 *Achillealigustica* All. H scap, W-Stenomedit.

C: Fringes

332 AnthemisarvensisL.subsp.arvensis T scap, Stenomedit.

C: Pastures

333 *Arctiumminus* (Hill) Bernh. H bienn, Eurimedit.

C: Fringes, clearings

334 BellisannuaL.subsp.annua T scap, Stenomedit.

C: Annual grasslands on wet soils

335 *Bellisperennis* L. H ros, Europ.-Cauc.

C: Wet meadows

336 *Bellissylvestris* Cirillo H ros, Stenomedit.

L: Perennial grasslands (lower altutides)

337 *Belliumbellidioides* L. H ros, Endem. Sa-Co-Bl-AT

C: Temporary ponds, wet soils

338 *Carlinacorymbosa* L. H scap, Stenomedit.

C: Pastures

339 CarthamuslanatusL.subsp.lanatus T scap, Eurimedit.

C: Pastures, nitrophilous vegetation near sheep pens

340 CentaureacalcitrapaL.subsp.calcitrapa H bienn, Eurimedit.

C: Pastures

341 *Chamaemelumfuscatum* (Brot.) Vasc. T scap, W-Stenomedit.

L: Temporary ponds

342 *Chondrillajuncea* L. H scap, S-Europ.-S-Sib.

C: Pastures

343 CichoriumintybusL.subsp.intybus H scap, Paleotemp.

L: Perennial grasslands

344 *Cirsiumscabrum* (Poir.) Bonnet & Barratte H scap, SW-Medit.

L: Fringes, road edges (lower altitudes)

345 Cirsiumvulgaresubsp.silvaticum (Tausch) Arènes H bienn, Eurimedit.

C: Fringes, road edges

346 *Crepisbellidifolia* Loisel. T scap, W-Stenomedit.

L: Pastures

347 *Crepisleontodontoides* All. H ros, W-Medit.-Mont.

C: Pastures

348 CrepisvesicariaL.subsp.vesicaria T scap, Submedit.-Subatl.

C: Pastures

349 *Crupinavulgaris* Cass. T scap, S-Sib.-Eurimedit.

L: Pastures, perennial grasslands

350 *Filagogallica* L. T scap, Eurimedit.

C: Annual grasslands

351 *Filagogermanica* (L.) Huds. T scap, Paleotemp.

U (S. Giorgio): Annual grasslands

352 *Galactitestomentosus* Moench H bienn, Stenomedit.

C: Pastures

353 *Glebioniscoronaria* (L.) Spach. T scap, Stenomedit.

L: Pastures, annual grasslands (lower altitudes)

354 Helichrysumitalicumsubsp.tyrrhenicum (Bacch., Brullo et Giusso) Herrando, J.M. Blanco, L. Sáez & Galbany Ch frut., Endem. Sa-Co-Bl

C: Garrigues

Notes: for this taxon, we follow Herrando-Moraira et al. (2016)

355 *Hyoserisradiata* L. H ros, Stenomedit.

C: Pastures, meadows

356 *Hypochaerisachyrophorus* L. T scap, Stenomedit.

C: Annual grasslands

357 *Hypochaeriscretensis* (L.) Bory & Chaub. H scap, NE-Medit.-Mont.

L: Dry pastures and rocky habitats

358 *Hypochaerisglabra* L. T scap, Eurimedit.

C: Pastures, meadows

359 HypochaerisradicataL.subsp.radicata H ros, Europ.-Cauc.

C: Pastures, meadows

360 *Hypochaerisrobertia* (Sch. Bip.) Fiori H ros, Endem. Sa-Co-Si-It

L: Wet rocks and cliffs

361 *Lactucamuralis* (L.) Gaertn. H scap, Europ.-Cauc.

C: Woods, fringes

362 *Leontodontuberosus* L. H ros, Stenomedit.

C: Grasslands, pastures

363 *Pilosellaziziana* (Tausch) F. W. Schultz & Sch. Bip. H scap, Europ. (?)

L: Grasslands

364 *Ptilostemoncasabonae* (L.) Greuter H scap, Endem. Sa-Co-AT-Hy

U (Entrance of the Domain): Road edge

365 *Pulicariaodora* (L.) Rchb. H scap, Eurimedit.

C: Woods, fringes (lower altitude)

366 *Reichardiapicroides* (L.) Roth H scap, Stenomedit.

L: Rocky habitats (lower altitudes)

367 *Rhagadiolusstellatus* (L.) Gaertn. T scap, Eurimedit.

C: Annual grasslands

368 ScolymushispanicusL.subsp.hispanicus H bienn, Eurimedit.

C: Pastures

369 SeneciovulgarisL.subsp.vulgaris T scap, Eurimedit.

C: Pastures, ruderal vegetation

370 Sonchusasper(L.)Hillsubsp.asper T scap, Euras.

C: Ruderal vegetation

371 *Sonchusoleraceus* L. T scap, Euras.

C: Ruderal vegetation

372 *Silybummarianum* (L.) Gaertn. H bienn, Medit.-Turan.

C: Ruderal vegetation, pastures

373 Taraxacumsect.Erythrosperma (H. Lindb.) Dahlst. or Taraxacumsect.Scariosa Hand.-Mazz. H ros, Circumbor.

C: Wet meadows

374 *Urospermumdalechampii* (L.) F.W. Schmidt H scap, Eurimedit.

C: Grasslands

**Dipsacales**

Adoxaceae

375 *Sambucusebulus* L. G rhiz, Eurimedit.

L: Streams

376 *Sambucusnigra* L. P caesp, Europ.-Cauc.

C: Woods, shrublands

Caprifoliaceae

377 *Dipsacusferox* Loisel. H bienn, Endem. Sa-Co-Itc

C: Pastures

378 *Valerianellaeriocarpa* Desv. T scap, Stenomedit.

C: Annual grasslands

**Apiales**

Araliaceae

379 *Hederahelix* L. P lian, Eurimedit.

C: Woods

Umbelliferae (*nom. altr.*Apiaceae)

380 *Buniumcorydalinum* DC. G bulb, Endem. Sa-Co

C: Garrigues, rocky habitats

381 *Chaerophyllumtemulum* L. T scap, Euras.

L: Woods, fringes

382 *Eryngiumcampestre* L. H scap, Eurimedit.

C: Pastures

383 FerulacommunisL.subsp.communis H scap, S-Eurimedit.

L: Pastures, clearings (lower altitudes)

384 *Oenanthecrocata* L. H scap, Medit.-Atl.

L: *Alnusglutinosa* woods, streams

385 *Oenanthelisae* Moris H scap, Endem. Sa

U (Funtana Arile spring): Wet meadows

Specimen examined: Funtana Arile, Anela, 08 June 1980, B. Corrias, S. Diana (SS)

386 *Oenanthepimpinelloides* L. H scap, Medit.-Atl.

C: Woods, fringes

387 *Saniculaeuropaea* L. H scap, Paleotemp.

C: Woods, fringes

388 Smyrniumperfoliatumsubsp.rotundifolium (Mill.) Bonnier & Layens H bienn, Stenomedit.

C: Fringes, woods

389 ThapsiagarganicaL.subsp.garganica H scap, S-Medit.

C: Pastures, grasslands

390 *Torilisafricana* Spreng. T scap, Medit.-Macaron.

C: Pastures, annual grasslands

391 *Torilisnodosa* (L.) Gaertn. T scap, Medit.-Turan.

C: Pastures, annual grasslands

## Ecological and biogeographical analysis of the indigenous flora of Anela

Here we assess the presence in the forest domain of Anela of 391 taxa, belonging to 32 orders and 74 families.

Of the listed taxa, 5 (*Anacamptislongicornu* (Orchidaceae), *Anchusahybrida* (Boraginaceae), Chenopodiumalbumsubsp.album (Amaranthaceae), *Dactylorhizainsularis* (Orchidaceae), *Scrophulariaumbrosa* (Scrophulariaceae)) were not found during our investigation. Excluding these species, then we recorded a total of 386 indigenous taxa within the domain. Two species are new for the Sardinian flora (*Agrostiscapillaris*, *Aspleniumadiantum-nigrum*) and, for 17 taxa, our findings determine an important enlargement of their known range on the island (Arrhenatherumelatiussubsp.sardoum, *Aspleniumforeziense*, Clinopodiumvulgaresubsp.orientale, *Colchicumnanum*, Danthoniadecumbenssubsp.decumbens, *Euphorbiasemiperfoliata*, *Exaculumpusillum*, Festucamorisianasubsp.morisiana, *Lamiummaculatum*, Mentharequieniisubsp.requienii, *Morisiamonanthos*, *Poabalbisii*, Prunusdomesticasubsp.insititia, Ranunculuscordigersubsp.cordiger, *Rosasubcanina*, Veronicavernasubsp.brevistyla, *Violareichenbachiana*).

Overall, we found 141 hemicryptophytes (36.1%), 137 therophytes (35.0%), 56 geophytes (14.3%), 27 phanaerophytes (6.9%), 15 nano-phanaerophytes (3.8%), 11 chamaephytes (2.8%), 3 hydrophytes (0.8%), and 1 helophyte (0.3%).

A total of 239 taxa belong to the Mediterranean element (61.1%), 53 are Eurasian *sensu lato* (including the true Eurasian, plus European, Euro-Siberian, Euro-Caucasian and Pontic district: overall 13.6%), 42 are Boreal-Temperate taxa (paleotemperate + circumboreal: 10.7%), 36 are widespread (cosmopolitan, sub-cosmopolitan and sub-tropical: 9.2%) and 19 are Atlantic (4.9%). We were not able to assign a geographical category to Prunusdomesticasubsp.insititia.

**Figure 2.**
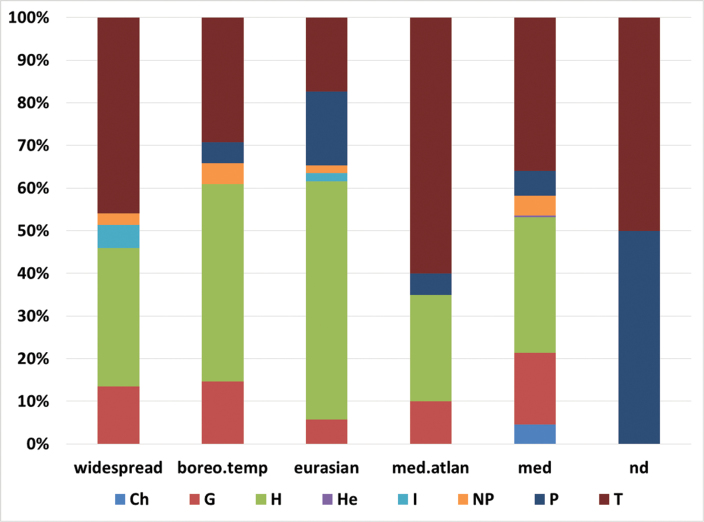
Percentage of biological types for each chorologic element detected in the vascular flora of Anela (390 taxa). boreo.temp = Boreal-temperate taxa; med.atlan = Mediterranean-Atlantic taxa; med = Mediterranean; nd = not determined.

Hemicryptophytes dominate within the Boreal-Temperate and the Eurasian components; annual species prevail within the widespread and the Mediterranean-Atlantic groups. The Mediterranean component hosts similar percentages of annuals and hemicryptophytes (Fig. 2).

The Mediterranean component is dominated by the euri-Mediterranean sub-element (94 taxa, 24.0% of the whole flora), followed by the steno-Mediterranean (77 taxa, 19.7%) and the endemics (45 entities, 11.5%). A total of 23 Mediterranean taxa belonged to other chorotypes (mountain-Mediterranean, Mediterranean-Turanian, Mediterranen-Macaronesian).

The endemic component of the flora of Anela is dominated by those of the Sardinian-Corsican biogeographic province (*sensu*Bacchetta et al. 2012) accounting for 28 taxa (endemics *sensu stricto*, 7.4%), of which 19 taxa are Sardinian-Corsican (42.2% of the endemic component), followed by Sardinian entities (5, 11.1%) and those present on Sardinia, Corsica and the Tuscan Archipelago (4, 8.9%). Tyrrhenian or Hercynian endemics (those present in Sardinia, Corsica, Tuscan Archipelago, the Balearic and Hyeres Islands and Sicily) account 12 (26.7%) and, finally, 11.1% is constituted by 5 entities with larger ranges including some continental areas (Sardinia and northern Africa or Sardinia and Italy).

**Figure 3.**
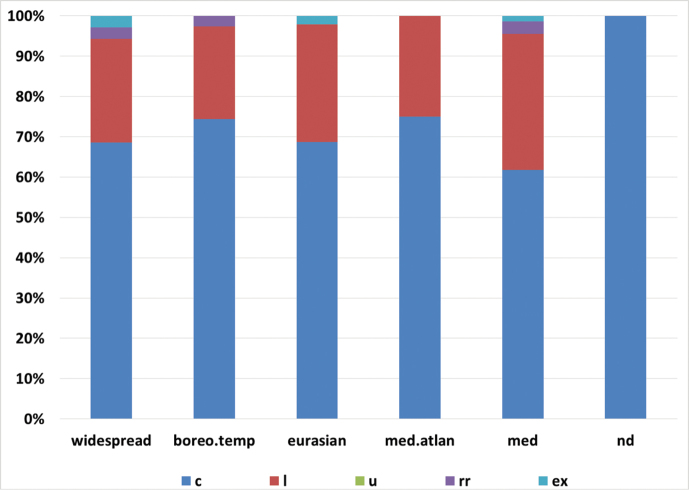
Percentage of abundance categories for each chorologic element detected in the vascular flora of Anela (390 taxa). c = common; l = localized; u = uncommon; rr = range restricted; ex = extinct. ; boreo.temp = Boreal-temperate taxa; med.atlan = Mediterranean-Atlantic taxa; med = Mediterranean; nd = not determined.

On the basis of our criteria, 241 taxa (61.6%) can be considered common at the local level, 113 (28.9%) are localised, 23 (5.9%) are uncommon, 9 (2.3%) are range restricted and 5 (1.3%) are locally extinct in the last 50 years. Common taxa are the dominant category in all the geographic groups, whereas range restricted taxa are found only in the widespread, Boreal-Temperate and the Mediterranean groups (Fig. 3).

A total of 176 out of 387 taxa were found mainly in grasslands habitats (45.5%) including dry pastures (61 taxa), annual and perennial grasslands (52 and 31 taxa, respectively) and wet pastures and meadows (32 taxa). Woodland habitats hosted 97 taxa (25.1%), comprising woods (57 taxa), fringes and clearings (30 taxa) and shrubs (10 taxa). Wet habitats (including *Alnusglutinosa* woods, springs, temporary ponds, ditches, muds, streams) hosted 53 taxa (13.7%). Rocky habitats (cliffs, rocks, screes) harbour 24 taxa (6.2%), then the garrigues hosted 21 taxa (5.4%) and finally the anthropogenic habitats (ruderal vegetation, buildings, walls, trampled sites, road edges) were the main habitat for 15 taxa (3.9%).

## Discussion

### Biogeographical description of the mountain

Our research discovered a high species density at the study area (30.6 taxa km^-2^), that is one of the highest ever documented in the Sardinian mountain floras (Table 1). Even if there is a clear inverse relationship between the area investigated and species’ density, we should note that, for areas having a comparable surface (~ 10 km^2^), the floristic density recorded at our study area is second only to the Mt. Gonare complex (Camarda 1984a, 1984b). It is noteworthy that the summit area of Sardinia (> 1500 m a.s.l.), having a surface of 16.8 km^2^, hosts “only” 214 taxa of which 66 are considered endemics (Arrigoni and Camarda 2015). So we can argue that areas at the edge between the Mediterranean and the temperate bioclimates, like Foresta Demaniale Anela and Mt. Gonare, host floristic components from both the two bioclimatic – biogeographic regions, having therefore more abundant floras than areas located in coastal or summit zones.

The hemicryptophytes/therophytes (H/T) ratio, as previously noted by Arrigoni and Camarda (2015), underlines the co-presence of two main elements, the perennial and the annual herbs, having very different life-cycles and summing 71.1% of our flora.The H/T ratio, that in Sardinia peaks at 2.5 at the summit of Gennargentu (Arrigoni and Camarda 2015), but decreases to 0.74 as the regional average, is at Anela 1.03. Limestone mountains like Mt. Albo, with a karst geology and consequently a pronounced summer drought, have a H/T ratio even lower than the regional average, whereas mountain complexes with impermeable substrates (plutonic, volcanic, metamorphic) approaching 1000 m a.s.l. have a H/T ratio ~ 1 gradually increasing with elevation (Table 1). This means that at 1000 m a.s.l., the co-presence of two large groups of non-woody plants, having an annual or perennial life cycle, has been detected: the annuals have a greater prevalence at lower altitudes, the perennials at higher altitudes and their ratio ~ 1 at 1000 m a.s.l. underlines the transition character of this altimetric level in Sardinia.

Important differences with the regional (Sardinian) value (Pignatti 1995) have also been detected for the Mediterranean floristic component, particularly the steno-Mediterranean taxa having a 28.9% regional percentage and 19.7% at the Anela forest domain; contrarily, the euri-Mediterranean component has 16.1% regional average but increases to 24% at our study area, the same percentage (24.3%) reached by the sum of the Boreal-Temperate and the Eurasian floristic components. Whereas lower altitude floras have a dominant steno-Mediterranean component and the floras at the summit of Mediterranean mountains show the prevalence of southern-European and Mediterranean orophytes and narrow endemics (Cañadas et al. 2014; Arrigoni and Camarda 2015), our flora is a good example of transition areas, having the 80% of taxa quite equally distributed amongst steno-Mediterranean, euri-Mediterranean, Boreal-Temperate and Eurasian and the endemic contingents. High species density, H/T ratio ~ 1, balance amongst different chorologic groups and endemic percentage ~ 10% can be considered characteristic features of mountain areas at the transition between the Mediterranean and the temperate bioclimates.

The composition of the flora of the Forest Domain of Anela is also peculiar because it is one of the few examples, not only in Sardinia but in the whole Mediterranean area, with no native Gymnosperms. Junipers (Juniperusphoeniceasubsp.turbinata (Guss.) Nym. and J.oxycedrussubsp.macrocarpa (Sibth. & Sm.) Neilr.) in NW Sardinia are mainly confined in coastal areas (Farris et al. 2017), but Yew (*Taxusbaccata* L.) and Prikly Juniper (JuniperusoxycedrusL.subsp.oxycedrus) are usually present in high hills and mountains. However junipers are not present in NW Sardinia inland areas (Farris et al. 2017), but the Yew is occurring in all the massifs and mountain ranges, including the two forest domains bordering Anela, the Fiorentini Forest Domain to the east (municipality of Bultei) and the Mt. Pisanu Forest Domain to the west (municipality of Bono, see Farris and Filigheddu 2008). The total absence of Gymnosperms in the native flora of the Anela forest domain is therefore surprising, most probably anomalous and it seems likely to be linked to the management history of the area rather than a natural pattern (Sechi and Falchi 2013).

Despite the fact that in 2004 (last forest census) 90.4% of the domain area was covered by forest or shrub communities (Sechi and Falchi 2013), it is striking that the 45% of the detected taxa were linked mainly to herbaceous habitats (annual and perennial grasslands, dry and wet pastures and meadows), already described for their peculiar and original floristic composition (Farris et al. 2013). Traditional grazing, particularly ovine pastoralism characterised by low flock density and transhumance, has been proven to be beneficial for the plant biodiversity of Mediterranean silvo-pastoral systems, whereas abandonment is detrimental even at short temporal scales (Farris et al. 2010a). The forest domain of Anela is a typical case where ovine stocks had a dramatic decrease in a short period: between 1990 and 2007, a decrease from 0.77 sheep ha^-1^ to 0.13 sheep ha^-1^ has been recorded (-83%, Farris et al. 2010a), whereas wood and shrub communities linked to potential natural vegetation (*sensu*Farris et al. 2010b) are recovering very fast, following a trend common to all Italy (Falcucci et al. 2007) and particularly to Sardinia (Puddu et al. 2012).

**Table 1.** Synthetic data on mountain floras from Sardinia and the regional flora, based on different sources (see notes below).

Site	Altitudinal interval	Area (km^2^)	No. taxa	Taxa / km^2^	H/T	No. endemics	% endemics	Source
Anela forest domain	600–1158	12.8	391	30.6	1.03	45	11.5	This work
Gennargentu	1500–1834	16.8	214	12.7	2.5	66	30.8	Arrigoni and Camarda 2015
Gennargentu	1000–1834	240	675	2.8	1.25	105	15.6	Arrigoni and Camarda 2015
Gennargentu	1000–1834	500	897^†^	1.8	1.03^‡^	n.d.	28^§^	Bacchetta et al. 2013
Supramontes	0–1463	335	n.d.	n.d.	n.d.	138	30 ^§^	Fenu et al. 2010
Mt. Albo	900–1127	68	659	9.7	0.61	48	7.3	Camarda 1984a
Mt. Gonare	538–1083	10	520	52	0.85	23	4.4	Camarda 1984b
Mt. Limbara	160–1359	166.24	923	5.5	0.75	80	8.7	Calvia and Ruggero unpublished
Mt. Limbara	800–1359	49.46	687	13.9	0.84	72	10.5	Calvia and Ruggero unpublished
Mt. Limbara	500–1359	n.r.	506	n.d.	1.18	55	10.9	Veri and Bruno 1974
Sardinia	0–1834	24090	2028	0.084	0.70	n.d.	7.1	Pignatti 1995
Sardinia	0–1834	24090	2400	0.099	n.d.	n.d.	n.d.	Arrigoni (2006–15)
Sardinia	0–1834	24090	2408^|^	0.1	0.74^¶^	290^#^	12	Various (see notes)
Sardinia	0–1834	24090	2149	0.09	n.r.	290	13.5	Médail 2017, table 2
Sardinia	0–1834	24090	2301	0.095	n.r.	331	14.4	Bartolucci et al. 2018

^†^Bacchetta et al. (2013) list 948 entities, including 10 varieties, 3 hybrids and 38 aliens: here we therefore consider 897 native taxa;

^‡^calculated by Arrigoni and Camarda 2015;

^§^Cañadas et al. 2014;

^|^Conti et al. 2005;

^¶^Arrigoni and Camarda 2015;

^#^Fenu et al. 2014; n.r. not reported; n.d. not determined.

### Conservation issues of this Flora

Even if rarity is not always linked to threat (de Lange and Norton 1998, Bacchetta et al. 2012), it is an important feature to consider when setting conservation priorities within long lists of taxa (Bacchetta et al. 2012, Le Berre et al. 2018), as in the case of the flora of the Anela forest domain. Additionally, 14 out of 32 uncommon and range-restricted taxa found in this flora are linked to wet habitats: some belong to the Mediterranean and endemic contingents (Cerastiumligusticumsubsp.palustre, *Exaculumpusillum*, *Isoeteshystrix*, Menthasuaveolenssubsp.insularis, Mentharequieniisubsp.requienii, *Morisiamonanthos*, *Oenanthelisae*), others to the Eurasian and Boreal-Temperate contingents (*Struthiopterisspicant*, *Carexremota*, *Irispseudacorus*, *Solanumdulcamara*, *Spiranthesspiralis*). Those habitats are supposed to be highly vulnerable (Filipe et al. 2013), as changes in land use and modification of water balance (because of climate change or human use) are amongst the most important threats to wetlands. Moreover, little is known about the resilience of associated plant communities, a threat increased by the high spatial isolation of such places within a Mediterranean context. At the study site, we detected several species having a contraction of range or local extinctions caused by the capture of surface or underground water for human use, as for example *Struthiopterisspicant*, Cerastiumligusticumsubsp.palustre, Mentharequieniisubsp.requienii and the localized fern *Osmundaregalis* for which we documented a local decrease > 50% in the last 20 years. Other species had a decrease directly caused by drainage of temporary ponds (*Exaculumpusillum*, *Isoeteshystrix*, *Morisiamonanthos*). Water management in a climatic changing scenario is and will increasingly be a key issue for the conservation of biodiversity in the Mediterranean basin (Casazza et al. 2014), a climatic change hotspot at the global scale (Giorgi 2006, Giorgi and Lionello 2008), where wet habitats and the species linked are amongst the most threatened (Ghosn et al. 2010, Pérez-Luque et al. 2015).

The 5 taxa, locally extinct, have no relationship with a particular habitat or human use from which they are (were) dependent for their survival in the area, with the exception of *Chenopodiumalbum* whose disappearance could be explained with the above-mentioned abandonment of pastoral activities, as it is a nitrophilous species. Their disappearance in the last decades, inferred from herbarium records, can be therefore a normal turnover in the composition of the local indigenous flora or an artifact derived from our sampling method (in the sense that these taxa are maybe still present in the area but we were not able to find them during our monthly sampling excursions).

Amongst the flora we inventoried, it is worth mentioning that several populations represent peripheral populations regarding the overall distribution of the taxa. First, a group of uncommon or range restricted species in the domain, are common plants in the Mediterranean bioclimate areas of Sardinia and sometimes in the whole basin. They are here confined to warm niches in the mountain area under study (*Anemonehortensis*, *Arbutusunedo*, *Arisarumvulgare*, *Arumpictum*, *Celtisaustralis*, *Ficuscarica*, *Ptilostemoncasabonae*), places relatively scattered through this mountain landscape. Oppositely, several Boreal-Temperate and Eurasian taxa confined in this sub-Mediterranean bioclimate island represent peripheral populations isolated sometimes by over 1000 km of their northern range. Those constitute rear edge populations (Hampe and Petit 2005) which may contain unique genetic variation, inherited from ancient species distribution and particular ecological conditions. These two contrasted situations have been highlighted several times within the Mediterranean flora (Lavergne et al. 2005, 2006) and are characteristic of those climatic transition areas. These plants all share the characteristic of occurring as fragmented, disjunct and often highly isolated populations, which restrain gene flow with central population (Pironon et al. 2017) and enhance amongst-population differentiation (Papuga et al. 2018). Thus, the relative isolation associated with potentially marginal ecological conditions highlight their evolutionary potential (Thompson 1999, Anacker and Strauss 2014), as it has recently been shown in Sardinia and Corsica for some marginal and peripheral populations of *Cyclamenrepandum* (Thompson et al. 2018). Additionally, these groups of taxa are often found in different macro-habitats which have very different links with human activities, therefore leading to different threats and management issues (Lavergne et al. 2006). Thus, conservation policies need to integrate such complex entities within their framework (Lesica and Allendorf 1995, Brunnell et al. 2004, Leppig and White 2006). Finally, those transition areas also contain numerous endemics, which render those places original and of high value for conservation.

Even if biodiversity hot-spots definition at multiple spatial scales is commonly based on the presence, density and distribution of endemic taxa (Myers et al. 2000, Cañadas et al. 2014), the data here presented support that other parameters should also be taken into account to more precisely define priority areas for conservation, as taxonomic complexity (Ennos et al. 2005) of floras and evolutionary potential of populations (Thompson et al. 2010), detected within continuous schemes of biodiversity monitoring (Marignani et al. 2014). This is particularly urgent in southern European mountains, whose biodiversity is threatened by both climate and land use changes (Bravo et al. 2008, Benito et al. 2011, Pauli et al. 2012, Vogiatzakis et al. 2016).
